# Robust adaptive control with lumped model uncertainty and wind disturbance estimation for airship trajectory tracking

**DOI:** 10.1371/journal.pone.0335392

**Published:** 2025-10-31

**Authors:** Muhammad Wasim, Ahsan Ali, Faisal Saleem, Inam Ul Hasan Shaikh, Jamshed Iqbal

**Affiliations:** 1 Department of Aeronautics and Astronautics Engineering, Institute of Space Technology, Islamabad, Pakistan; 2 Department of Electrical Engineering, University of Engineering and Technology, Taxila, Pakistan; 3 Department of Measurements and Control Systems, Silesian University of Technology, ul. Akademicka 16, Gliwice, Poland; 4 The Joint Doctoral School, Silesian University of Technology, ul. Akademicka 2A, Gliwice, Poland; 5 School of Digital and Physical Sciences, Faculty of Science and Engineering, University of Hull, United Kingdom; Vignan's Institute of Information Technology, INDIA

## Abstract

The robotic airship can be used as an aerostatic platform for many potential applications, for example, communication, hovering payload deliveries, data-gathering for research studies, etc. These applications require a fully autonomous perspective of an airship. One of the important aspects of airship autonomy is trajectory tracking control. An airship has complex and uncertain nonlinear dynamics which pose a major challenge for designing a precise trajectory tracking control. This paper addresses the airship trajectory tracking control problem under model uncertainties and wind disturbance. We propose a lumped model uncertainties and wind disturbance estimation approach based on an unscented Kalman filter. The estimated lumped uncertainty is used by the Sliding Mode Controller (SMC) for ultimate control of airship trajectory tracking. This comprehensive algorithm, Unscented Kalman filter-based Sliding Mode Controller (USMC), is used as a robust adaptive control solution to track the desired trajectory. The stability and convergence of the proposed method are investigated using the Lyapunov stability analysis. Simulation results show that the proposed method efficiently tracks the desired trajectory. The method solves the stability, convergence, and chattering problem of SMC without the bound constraint of model uncertainties and wind disturbance.

## 1. Introduction

Unmanned Aerial Vehicles (UAVs) design is the focus of researchers and developers worldwide owing to its numerous applications in agriculture and in the commercial sector [1]. UAVs cover various aerial vehicles, including fixed-wing aircraft, quadrotors, and airships [2]. The airship is a typical type of lighter-than-air vehicle that takes maximum airlift from aerostatic forces. The airship has a prominent future due to its unique properties and suitability for diverse potential applications. Since an airship uses minimum power to stay aloft, it can be operated for a prolonged period as a data collection platform. It can also provide a potential platform for agriculture monitoring at low altitudes for pests, weeds, and crop data collection.

These applications require autonomous tasks that include trajectory tracking and path following. Trajectory tracking ensures that an airship follows a predefined path at a specific time. Path following requires the airship to follow a particular predefined path without any time constraints. For successful execution of autonomous goals, the foremost requirement is to develop an efficient and reliable control and navigation system. Airship trajectory tracking control design is a challenging task due to its slow and highly coupled nonlinear dynamics. The difficulty level further increases due to its lighter-than-air nature because it is severely affected by wind disturbances. Model uncertainties related to mass matrix variation in the airship model also pose a great deal of challenges in control design. Furthermore, unknown parameters in the aerodynamic model affect performance of the controller. Many contributions can be found in the literature suggesting different control mechanisms for airship trajectory tracking.

## 2. Literature

In linear control methods, Proportional-Integral-Derivative (PID) controller has been designed for airship trajectory tracking. Chen et al. designed a composite control scheme for airship trajectory tracking which consists of PID and dynamic inversion controller for improved performance [3]. However, the PID controller cannot ensure control capability for different operating conditions. Moutinho et al. linearized the AURORA airship model and separated its longitudinal and lateral dynamics. Based on this reduced model, a dynamic inversion controller was implemented [4]. However, this control method does not apply to all operating points. This is because the controller neglects dynamic nonlinearities and coupling between longitudinal and lateral dynamics [5].

Apart from these methods, researchers have used Model Predictive Control (MPC) with the linear model for airship planar trajectory tracking. However, mechanisms for evaluating controllers under a stratospheric environment are not discussed. Moreover, too much dependence on operating space affects the performance of linear MPC. A nonlinear MPC is designed for airship spatial trajectory tracking while handling operating space limitations. MPC method is computationally complex because it solves an optimization problem on each sampling instant. The complexity of this algorithm further increases with each additional state and restrains its use in real-time scenarios. Literature also presents the nonlinear controllers, including Gain Scheduling Controller (GSC), Back Stepping Controller (BSC), and Sliding Mode Controller (SMC) for airships. In the project AURORA, airship model is linearized around different operating points and a GSC is synthesized for each vertex. They applied interpolation for the points between vertices to generate control efforts. The complexity of the controller synthesis increases with several operating points. Hence in some instances, the controller synthesis problem becomes infeasible. Therefore, many researchers have preferred other nonlinear control approaches for airship trajectory tracking [6–12].

A command-filtered vectorial BSC is designed for a stratospheric airship to track a 3D trajectory. The command filter is used to smoothen the derivatives of controller input for the backstepping approach which is employed to reduce the tracking error. This approach was able to achieve robustness when the gains of the controller were high. Although high controller gains ensure robust performance, it deteriorates nominal performance. In such cases, model deficiencies are handled through adaptive and neural network-based adaptive BSCs. It is reported that the performance of the adaptive backstepping algorithm strictly depends on initial bias and disturbances. If the initial bias and disturbances are significant, the method may result in system instability. The use of a BSC is problematic because of “explosion of terms” in its control law depending on the size of the system [13–18].

SMC is designed for airship planar trajectory tracking under the project AURORA, using linear longitudinal and lateral models. The longitudinal model is used to maintain a certain height of the airship and the lateral model is incorporated to reduce tracking error. A model used for the controller design was linearized for an airspeed of 2 $ms^{-\text{1}}$. So, this approach does not guarantee global behavior. Apart from that, fuzzy logic and neural networks-based SMC methods are also applied for airship trajectory tracking. These methods also estimate the model uncertainties and wind disturbance. The existing literature for airship trajectory tracking control based on adaptive or intelligent methods to handle model uncertainties and wind disturbance does not provide a generic solution to the airship trajectory tracking problem. Also, the authenticity and reliability of data are crucial to the training part of the algorithms operating based on neural network (NN). In case all scenarios of the workspace are not covered in the training data, the NN-based SMC will not perform for the missing scenarios. The data for missing scenarios needs to be estimated using some estimators [19–22].

The discontinuous nature of SMC and imperfections in the physical system induce high-frequency oscillations in control input known as chattering [23,24]. In airship control, chattering might damage the actuators. Moreover, the reaching phase of the controller is sensitive to model uncertainties and external disturbances. This degrades the performance of the controller in some instances and may cause instability. An airship model is usually affected by mass matrix variations, aerodynamic coefficients uncertainties, and wind disturbances. To handle model uncertainties and wind disturbances, this work presents a modified SMC method for airship trajectory tracking. Since this method can approximate the model uncertainties and wind disturbances, we mention this as an Adaptive SMC. An adaptive law is designed to provide the lumped model uncertainties and wind disturbance estimates to the SMC to minimize the chattering issue and ensure robustness.

### 3.1. Contribution of the proposed work

Estimating airship model uncertainties and wind disturbance is mandatory for efficient trajectory tracking. In most of the trajectory tracking control solutions, specified bounds are applicable. These bounds need to be relaxed for a global trajectory-tracking solution. In our previous work [25–28], we have proposed the airship aerodynamic model estimation and lumped model uncertainty estimation using Extended Kalman Filter (EKF) and Unscented Kalman Filter (UKF). In this research, we are extending our previous results and proposing airship trajectory tracking control using the Unscented Kalman filter-based SMC method. The proposed method provides a generic solution to airship trajectory tracking which relaxes the uncertainty bounds. The purpose of the proposed research is to introduce the Kalman filter algorithm as a lumped model uncertainty and wind disturbance estimator. The proposed method is simple, easy to understand and implement. For airship state estimation, the availability of off-the-shelf sensor fusion-based position, attitude, and linear and angular velocity estimations have been assumed. The proposed algorithm is tested under a generalized case where parameter variation and wind disturbance are considered at different time intervals during the airship aerodynamic flight. The simulation results show improved performance of the proposed USMC method over the conventional SMC. Equations of conventional SMC for airship trajectory tracking have also been derived. This work used conventional SMC for performance evaluation and comparison of the proposed approach.

Key contributions of this work include:

1. Mathematical modelling of an airship lumped with modelling uncertainties and wind disturbances.
2. Design of UKF-based estimator for lumped uncertainty estimation for airship.
3. A generic adaptive SMC approach for relaxed uncertainty bounds.
4. Attenuation of chattering introduced by SMC for an airship trajectory tracking.

## 3. Airship modeling

**Fig 1:**
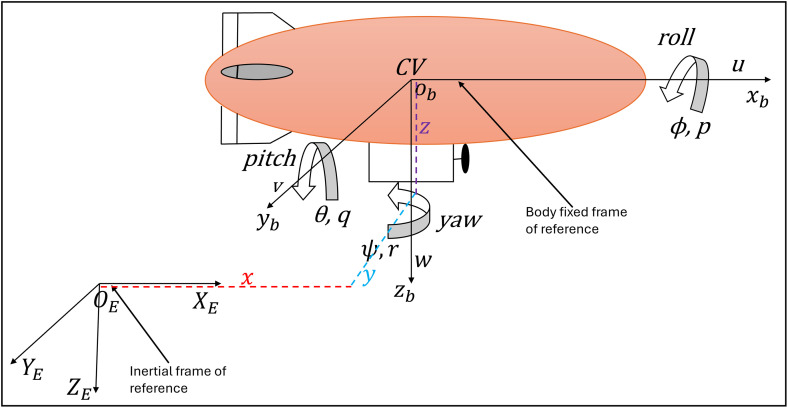
The coordinate system of an airship.

**Fig 2:**
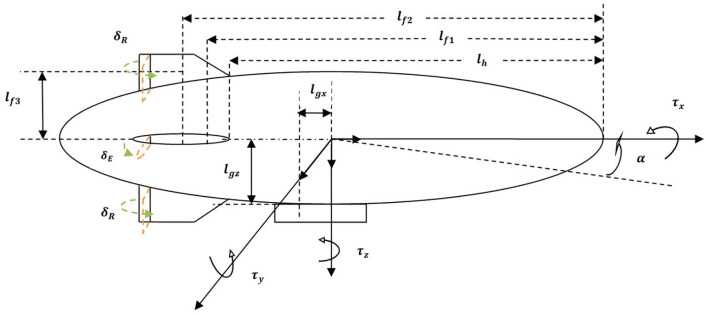
Geometrical parameters of ellipsoidal airship.

Fig 1 shows an airship which has an ellipsoidal envelope filled with low-density gas such as helium. The airship has a gondola under the envelope which carries autonomous flight control equipment, batteries, and payload. It has two propellers on both sides of the gondola to provide the necessary thrust for airship motion. There are two rudders and two elevators making a plus configuration on the tail. They provide the aero-dynamic control force necessary for airship maneuvering in cruise flights. A propeller at the bottom rudder of the airship enhances yaw control. Fig 2 shows the geometrical parameters of the airship. The introduction of two reference frames enables the development of airship nonlinear modelling equations. The inertial frame is located at a fixed point on Earth. The local frame of reference is located at the Center of Volume (CV) of the airship [28].

Airship position is expressed through the inertial frame ($O_{E}X_{E}Y_{E}Z_{E}$) and represented byX=[x,y,z]. Here, x is the distance covered by airship in x-direction, y is the distance covered in y-direction and z is the distance covered in upward direction. Θ=[φ,θ,ψ] denotes attitude in inertial frame. Where, φ,θ,ψ represent the roll, pitch, and yaw angle with respect to inertial frame ($O_{E}X_{E}Y_{E}Z_{E}$). Airship linear and angular velocities aligned with the local frame of reference ($O_{b}X_{b}Y_{b}Z_{b}$) are represented by υ=[u,v,w] and Ω=[p,q,r], respectively. $X_{I}=[\begin{array}{cc} X & \Theta \end{array}]$ is a state vector representing airship position and attitude. $V_{b}=[\begin{array}{cc} \upsilon & \Omega \end{array}]$ is a state vector representing airship linear and angular velocities. Equation (1) is a nonlinear state-space representation of the airship model with nominal parameters. Whereas equation (2) represents the airship model with uncertain parameters. Uncertainty and wind disturbance are represented with lumped uncertainty vector$\;d_{l}$.


$$\left\{ \begin{array}{c} \begin{array}{c} \begin{array}{c} {\dot{X}}_{I}=R(\Theta )V_{b}\\ {\dot{V}}_{b}=M^{-\text{1}}\left(F_{d}+F_{As}+F_{Ad}+U\right) \end{array} \end{array} \end{array}\right.\;$$ (1)

$$\left\{ \begin{array}{c} \begin{array}{c} \begin{array}{c} {\dot{X}}_{I}=R(\Theta )V_{b}\\ {\dot{V}}_{b}=M^{-\text{1}}\left(F_{d}+F_{As}+F_{Ad}+U\right)+d_{l} \end{array} \end{array} \end{array}\right.\;$$ (2)

where,


$$d_{l}=(\Delta M+M{)}^{-\text{1}}\left(\left(\Delta F_{d}+F_{d}\right)+\left(\Delta F_{As}+F_{As}\right)+\left(\Delta F_{Ad}+F_{Ad}\right)+F_{w}+U\right)-M^{-\text{1}}\left(F_{d}+F_{AS}+F_{Ad\;}+U\right)$$

R(Θ) is the direction cosine matrix. It converts airship linear and angular velocities from body axis to inertial frame, rate of change of airship position and attitude. $F_{d}$ is the dynamic force vector. It represents the forces and torques acting on airship due to centrifugal and Coriolis affects. $F_{As}$ is the aerostatic force vector; forces and torques acting on airship due to gravity and buoyancy. $F_{Ad}$ are the aerodynamic forces and torques acting on airship; they consist of drag, lift, and side force. U is the generalized control input vector. The notations $M,\;F_{d},\;F_{As},\;F_{Ad}$ are used for the nominal model while $\Delta M,\;\Delta F_{d},\;\Delta F_{As},\;\Delta F_{Ad}$ encapsulate the parameter variations. The mass matrix is:


$$M=[\begin{array}{cccccc} m_{x} & \text{0} & \text{0} & \text{0} & m_{\text{1}\text{5}} & \text{0}\\ \text{0} & m_{y} & \text{0} & m_{\text{2}\text{4}} & \text{0} & m_{\text{2}\text{6}}\\ \text{0} & \text{0} & m_{z} & \text{0} & m_{\text{3}\text{5}} & \text{0}\\ \text{0} & m_{\text{4}\text{2}} & \text{0} & J_{x} & \text{0} & {-J}_{xz}\\ m_{\text{5}\text{1}} & \text{0} & m_{\text{5}\text{3}} & \text{0} & J_{y} & \text{0}\\ \text{0} & m_{\text{6}\text{2}} & \text{0} & {-J}_{xz} & \text{0} & J_{z} \end{array}]$$ (3)

The mass matrix contains the actual mass, inertia, the added-mass and inertia terms for the airship. The added terms come due to the mass of air displaced by the airship. Equation (4) describes the dynamic force vector. The dynamic force vector summarizes the forces and torques acting on the airship due to centrifugal and Coriolis effects.


$$F_{d}=[\begin{array}{c} -m_{z}wq+m_{y}rv+m\left[a_{x}\left(q^{\text{2}}+r^{\text{2}}\right)-a_{z}rp\right]\\ -m_{x}ur+m_{z}pw+m\left[-a_{x}pq-a_{z}rq\right]\\ -m_{y}vp+m_{x}qu+m\left[-a_{x}rp+a_{z}\left(q^{\text{2}}+p^{\text{2}}\right)\right]\\ -\left(J_{z}-J_{y}\right)rq+J_{xz}pq+ma_{z}(ur-pw)\\ -\left(J_{x}-J_{z}\right)pr+J_{xz}\left(r^{\text{2}}-p^{\text{2}}\right)+\ldots \\ m\left[a_{x}(vp-qu)-a_{z}(wq-rv)\right]\\ -\left(J_{y}-J_{x}\right)qp-J_{xz}qr+m\left[-a_{x}(ur-pw)\right] \end{array}]$$ (4)

$F_{As}$ and $F_{Ad}$ represent the aerostatic and aerodynamic force vectors as:


$$F_{As}=[\begin{array}{c} -\left(W-B_{f}\right)s_{\theta }\\ \left(W-B_{f}\right)c_{\theta }s_{\varphi }\\ \left(W-B_{f}\right)c_{\theta }c_{\varphi }\\ a_{z}Wc_{\theta }s_{\varphi }\\ -{(a}_{z}W-b_{z}B_{f})s_{\theta }-{(a}_{x}W-b_{x}B_{f})c_{\theta }c_{\varphi }\\ a_{x}Wc_{\theta }s_{\varphi } \end{array}]$$ (5)

$$F_{Ad}=f\left(V_{t}\right)[\begin{array}{c} C_{X\text{1}}c_{\alpha }^{\text{2}}c_{\beta }^{\text{2}}+C_{X\text{2}}s_{\text{2}\alpha }s_{\frac{\alpha }{\text{2}}}\\ C_{Y\text{1}}c_{\frac{\beta }{\text{2}}}s_{\text{2}\beta }+C_{Y\text{2}}s_{\text{2}\beta }+C_{Y\text{3}}s_{\beta }s_{\left|\beta \right|}\\ C_{z\text{1}}c_{\frac{\alpha }{\text{2}}}s_{\text{2}\alpha }+C_{z\text{2}}s_{\text{2}\alpha }+C_{z\text{3}}s_{\alpha }s_{\left|\alpha \right|}\\ C_{L\text{2}}s_{\beta }s_{\left|\beta \right|}\\ C_{M\text{1}}c_{\frac{\alpha }{\text{2}}}s_{\text{2}\alpha }+C_{M\text{2}}s_{\text{2}\alpha }+C_{M\text{3}}s_{\alpha }s_{\left|\alpha \right|}\\ C_{N\text{1}}c_{\frac{\beta }{\text{2}}}s_{\text{2}\beta }+C_{N\text{2}}s_{\text{2}\beta }+C_{N\text{3}}s_{\beta }s_{\left|\beta \right|} \end{array}]$$ (6)

were, W is the airship weight, $B_{f}$ is the buoyancy force*, {*$a_{x},a_{z}$*}* are the coordinates of the Center of Gravity (CG) of the airship, and *{*$b_{x},\;b_{z}$*}* are the coordinates of the Center of Volume (CV) of the airship*.*

Also,


$$f\left(V_{t}\right)=\frac{\text{1}}{\text{2}}\rho V_{t}^{\text{2}}$$

$$V_{t}=\sqrt{{(u}^{\text{2}}+v^{\text{2}}+w^{\text{2}})}$$

and α denotes the angle of attack while $C_{ij}(i=X,Y,Z,L,M,N;j=\text{1},\text{2},\text{3},\text{4})$ represent the aerodynamic coefficients.

The details of parameters are:

**Table:** 

V	:	Airship volume
g	:	Gravitational acceleration
H	:	Airship heaviness value (it is zero for neutral buoyancy case)
$J_{x}$	:	Moment of inertia about x-axis
$J_{y}$	:	Moment of inertia about y-axis
$J_{z}$	:	Moment of inertia about z-axis
$J_{xz}$	:	Moment of inertia about xz-axis
l	:	Length of airship
d	:	Airship maximum diameter
$B_{f}$	=	ρgV
W	=	B+Hg
$m_{x}$	=	$m-X_{\dot{u}}$
$m_{y}$	=	$m-Y_{\dot{v}}$
$m_{z}$	=	$m-Z_{\dot{w}}$
$m_{\text{1}\text{5}}$	=	${ma}_{z}-X_{\dot{q}}$
$m_{\text{2}\text{4}}$	=	${-ma}_{z}-Y_{\dot{p}}$
$m_{\text{2}\text{6}}$	=	${ma}_{x}-Y_{\dot{r}}$
$m_{\text{3}\text{5}}$	=	$-{ma}_{x}-Z_{\dot{q}}$
$m_{\text{4}\text{2}}$	=	$-{ma}_{z}-L_{\dot{v}}$
$m_{\text{5}\text{1}}$	=	${ma}_{z}-M_{\dot{u}}$
$m_{\text{5}\text{3}}$	=	${-ma}_{x}-M_{\dot{w}}$
$m_{\text{6}\text{2}}$	=	${ma}_{x}-N_{\dot{v}}$
$Z_{\dot{w}}$	=	$Y_{\dot{v}}$
$N_{\dot{r}}$	=	$M_{\dot{q}}$
$\overset{―}{m}$	=	B/g
${\overset{―}{I}}_{y}$	=	$\frac{\overset{―}{m}(l^{\text{2}}+d^{\text{2}})}{\text{2}\text{0}}$

The origin of the body frame is located at the center of volume of the airship. For the double-ellipsoid geometry, the center of volume is located on the x-axis at the point $x_{cv}$. These parameters are adopted from [27–31].

**Remark 1.** Airship roll and pitch angles satisfy the bounds $\left\{|\varphi |<\frac{\pi }{\text{2}};|\theta |<\frac{\pi }{\text{2}}\right\}$. This ensures non-singularity of the rotation matrix.

**Assumption 1.** The Airship CG point lies beneath the CV and its center of buoyancy coincides with the CV. The airship is in a neutral buoyancy state such that its weight and buoyancy are equal. Consequently, the aerostatic forces do not affect horizontal dynamics. The given 6-DOF equations ignore the aeroelastic effects and consider the airship as a rigid body. However, the controller design considers these effects as model uncertainties.

**Assumption 2.** Airship mass matrix terms are uncertainty terms:


$$m_{x}=m_{x\text{0}}+m_{x\Delta }$$

$$m_{y}=m_{y\text{0}}+m_{y\Delta }$$

$$m_{z}=m_{z\text{0}}+m_{z\Delta }$$

$$J_{x}=J_{x\text{0}}+J_{x\Delta }$$

$$J_{y}=J_{y\text{0}}+J_{y\Delta }$$

$$J_{z}=J_{z\text{0}}+J_{z\Delta }$$

$$J_{xz}=J_{xz\text{0}}+J_{xz\Delta }$$

where $m_{x\text{0}}$*,* $m_{y\text{0}}$*,* $m_{z\text{0}}$*,* $J_{x\text{0}}$*,* $J_{y\text{0}}$*,* $J_{z\text{0}}$*,* $J_{xz\text{0}}$ are known and $m_{x\Delta }$*,* $m_{y\Delta }$*,* $m_{z\Delta }$*,* $J_{x\Delta }$*,* $J_{y\Delta }$*,* $J_{z\Delta }$*,* $J_{xz\Delta }$*,* are unknown parts*.* However, the unknown parts are bounded by some upper bound; ${\overset{―}{m}}_{x\Delta }$*,* ${\overset{―}{m}}_{y\Delta }$*,* ${\overset{―}{m}}_{z\Delta }$*,* ${\overset{―}{J}}_{x\Delta }$*,* ${\overset{―}{J}}_{y\Delta }$*,* ${\overset{―}{J}}_{z\Delta }$*,* ${\overset{―}{J}}_{xz\Delta }$*,* ${\overset{―}{m}}_{ij\Delta }$*.* The aerodynamic model coefficients $C_{ij}=C_{ij\text{0}}+C_{ij\Delta }\;(i=X,Y,Z,L,M,N;j=\text{1},\text{2},\text{3},\text{4})$ are uncertain*.* Where $C_{ij\text{0}}$ is a known part, and $C_{ij\Delta }$ is an unknown part. The unknown part is bounded by some upper-bound $\;{\overset{―}{C}}_{ij\Delta }$.

## 4 Controller design for trajectory tracking

Trajectory tracking control is a task of tracking a predefined time-varying reference path within a specific time. Let $X_{Id}$, $X_{I}$ represent the reference and actual trajectories, respectively, where,


$$X_{Id}={\left[{\begin{array}{cccccc} x_{d} & y_{d} & z_{d} & \varphi_{d} & \theta_{d} & \psi \end{array}}_{d}\right]}^{\text{T}}$$

and


$$X_{I}=[\begin{array}{cccccc} x & y & z & \varphi & \theta & \psi \end{array}{]}^{\text{T}}$$

**Fig 3:**
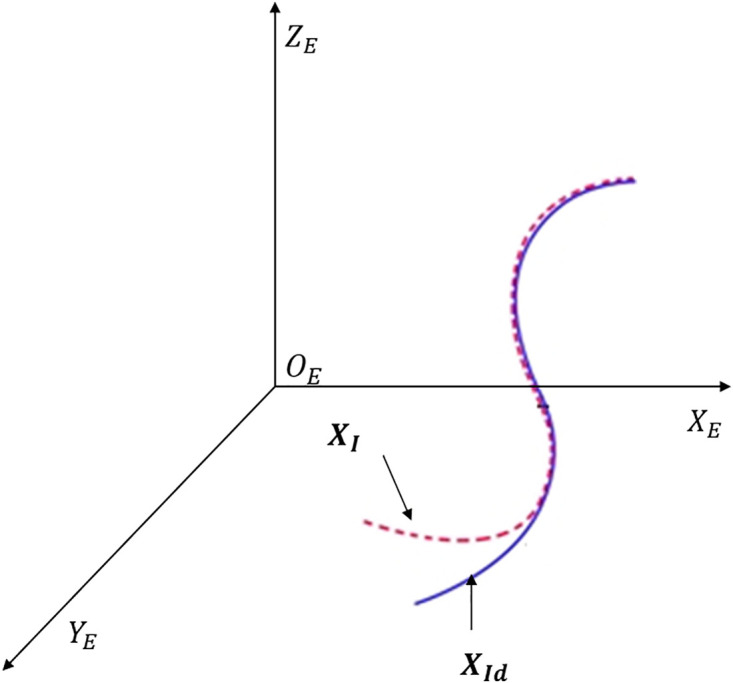
Visual representation of a typical trajectory tracking problem.


The trajectory tracking controller aims to bring the error, i.e., $x_{e}=X_{I}-X_{Id}$, equal to zero asymptotically. Fig 3 gives a visual representation of a trajectory-tracking problem. Tracking error formulation is:

$$\underset{t\to \infty }{\text{lim}}x_{e}=\underset{t\to \infty }{\text{lim}}\left|X_{I}-X_{Id}\right|=\text{0}$$ (7)

**Fig 4:**
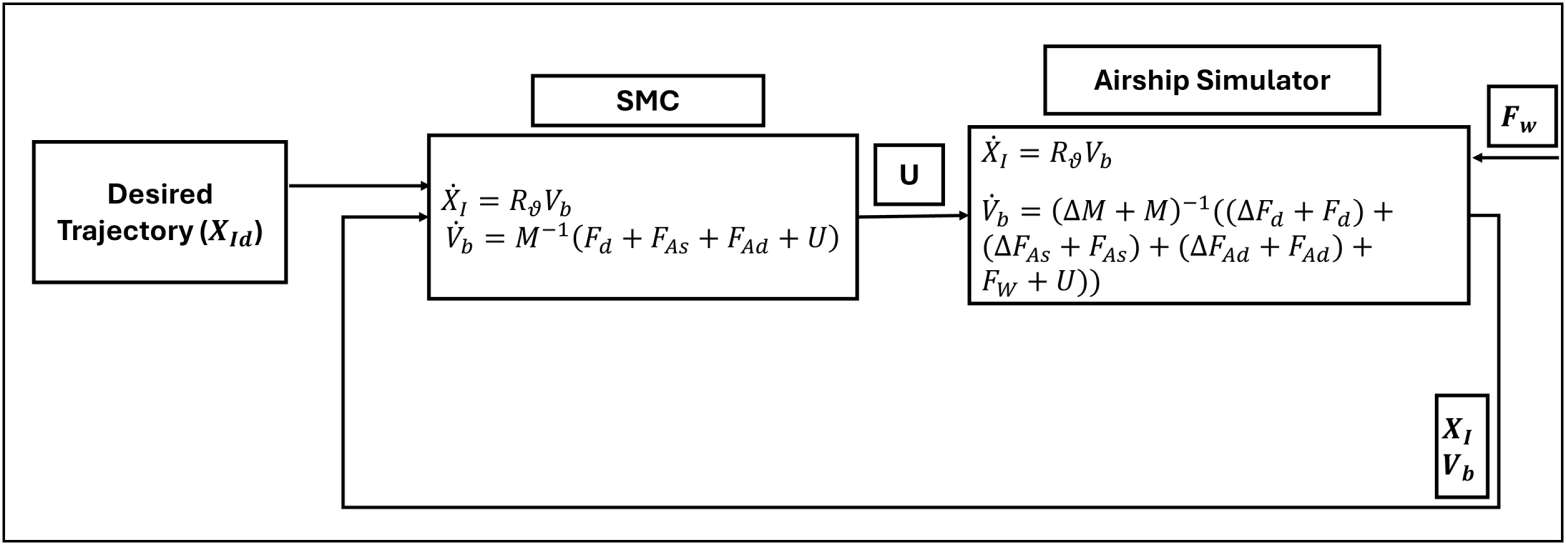
The block diagram for the sliding mode controller.

In this research, a sliding mode controller is used as a benchmark for comparing the results with the proposed USMC method. Therefore, for conventional SMC, we have used the model in equation (1) for controller design. Fig 4 demonstrates the models which we use in the controller and the simulator. In the simulator, we can introduce the parameter variation during the simulation run to mimic the actual environment and parameter variation scenarios. This allows to validate the controller’s performance under model uncertainties and wind disturbance. In the simulator, Δ used with vectors $F_{d}$, $F_{As}$, $F_{Ad}$ and mass matrix M, shows that the parameters of the model are varied during the simulation run.

The tracking error is:


$$x_{e}=X_{I}-X_{Id}$$ (8)

Let us define the error-based linear hyperplane as a sliding surface:


$$S={\dot{x}}_{e}+ax_{e}$$ (9)

where, $a=\text{ diag}\left(a_{\text{1}},a_{\text{2}},a_{\text{3}},a_{\text{4}},a_{\text{5}},a_{\text{6}}\right)$. These terms are tuning variables and must be positive to ensure the convergence of error to zero. After the design of the sliding surface, this work chooses the appropriate reaching law. The reaching law ensures that trajectories converge to the sliding surface and stay there. Here, the simplest reaching law “−Ksign(S)” is selected as a switching function. Where K=diag$\left(k_{\text{1}},k_{\text{2}},k_{\text{3}},k_{\text{4}},k_{\text{5}},k_{\text{6}}\right)$ denotes the controller gains. The gains are positive and can be tuned heuristically; however, as for SMC, the uncertain term $d_{l}$ is not available. For robustness to prevail, assumption 3 should hold for conventional SMC.

**Assumption 3.** It is assumed that there exists a positive number γ such that the uncertainty $\left\|d_{l}\right\|<\gamma$, and controller gain ‖K‖>γ.

For the trajectories to reach the sliding surface, the reachability condition should be satisfied, i.e., $S\dot{S}<\text{0}.$ for the validity of this condition, $\dot{S}$ is selected as -Ksign(S). Where ‘*sign’* is the signum function which is zero at S=0, one for S>0, and a negative one at S<0. Now, using equation (1) with the availability of bounds on $d_{l}$, as defined in assumption 3 and the reachability condition, the control law for conventional SMC is:


$$U=M{R(\theta )}^{-\text{1}}\left(-K\text{sign}(S)-\dot{R}(\theta )V_{b}+{\ddot{X}}_{Id}-a{\dot{x}}_{e}\right)-\left(F_{d}+F_{Ad}+F_{As}\right)$$ (10)

**Theorem 1.** The airship model given in equation (1) with the sliding surface of equation (9), the control law in equation (10) brings the trajectory tracking error asymptotically to zero.

**Proof.** Selecting the Lyapunov function as:


$$V=\frac{\text{1}}{\text{2}}S^{T}S>\text{0}$$ (11)

The existence of the Lyapunov function proves the existence condition for sliding modes. V is the Lyapunov function concerning S, i.e., the sliding surface. Since *V* (0) = 0, as S = 0, and $V=\text{1}/\text{2}\;S^{T}S>\text{0}$, this is a positive definite Lyapunov function. S = 0 is the equilibrium point to which the state is to converge. ‘State’ is $x_{e}$, which is the error between the system state and the desired state, i.e., $x_{e}\;=\;X_{I}\;-\;X_{d}$. The objective of SMC is to bring the tracking error to zero. At S = 0, the system will be on the sliding surface, and the error dynamics will reduce to:


$$x_{e}(t)=x_{e}(\text{0})e^{-at}$$ (12)

Equation (13) is derived by differentiating (11) and expanding the terms.


$$\dot{V}=S^{T}\dot{S}=S^{T}\left[{\ddot{x}}_{e}+a{\dot{x}}_{e}\right]$$ (13)

Differentiating equation (8) twice results in:


$${\ddot{x}}_{e}={\ddot{X}}_{I}-{\ddot{X}}_{\text{1}d}$$ (14)

From equation (1), we have:


$${\ddot{x}}_{e}=\dot{R}(\Theta )V_{b}+{R(\Theta )\dot{V}}_{b}-{\ddot{X}}_{Id}=\dot{R}(\Theta )V_{b}+{R(\Theta )M}^{-\text{1}}\left(F_{D}+F_{AS}+F_{AD}+U\right)+R(\Theta )d_{l}-{\ddot{X}}_{Id}$$ (15)

Substitution of equation (15) into equation (13) results in:


$$\dot{V}=S^{T}[\dot{R}(\Theta )V_{b}+{R(\Theta )M}^{-\text{1}}\left(F_{D}+F_{AS}+F_{AD}+U\right)+R(\Theta )d_{l}-{\ddot{X}}_{Id}+a{\dot{x}}_{e}]$$ (16)

Substituting control law in equation (16) and simplifying results in:


$$\dot{V}=-S^{T}\left[K\text{sign}(S)-R(\Theta )d_{l}\right]$$ (17)

Lyapunov condition applies to the inequality ‖K‖>γ.


$$\dot{V}=-S^{T}\left[K\text{sign}(S)-R(\Theta )d_{l}\right]<\text{0},\;(S(t\neq \text{0}))$$ (18)

**Remark 2.** The SMC controller in equation (10) ensures the Lyapunov stability of the airship. The trajectory tracking error will asymptotically converge to zero within a finite time for the given bounds.

### 4.1 USMC design for airship trajectory tracking

The bounds given in assumption 3 undermines the performance of the sliding mode controller. Estimation of model uncertainty and wind disturbance will ensure the performance of SMC beyond the given bounds. An unscented Kalman filter estimates model uncertainties and wind disturbance in the proposed algorithm.

**Fig 5:**
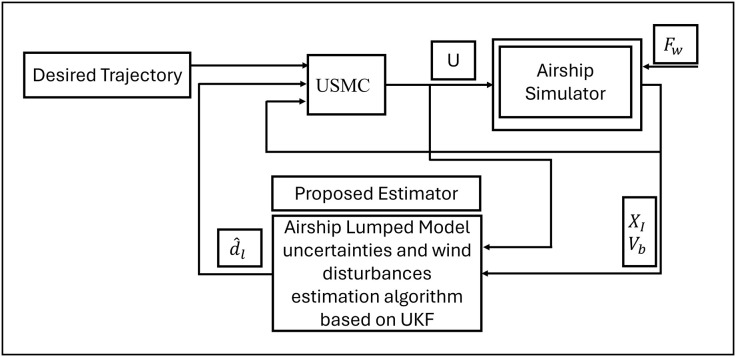
Block diagram representation of proposed USMC algorithm.

Fig 5 shows the block diagram of the proposed Unscented Kalman filter-based Sliding Mode Controller (USMC). SMC is provided with the reference trajectory and the estimate of airship states, model uncertainties, and wind disturbance. The SMC calculates the control input and provides the airship system. In response to the control input, the airship tracks the desired trajectory effectively.

**Fig 6:**
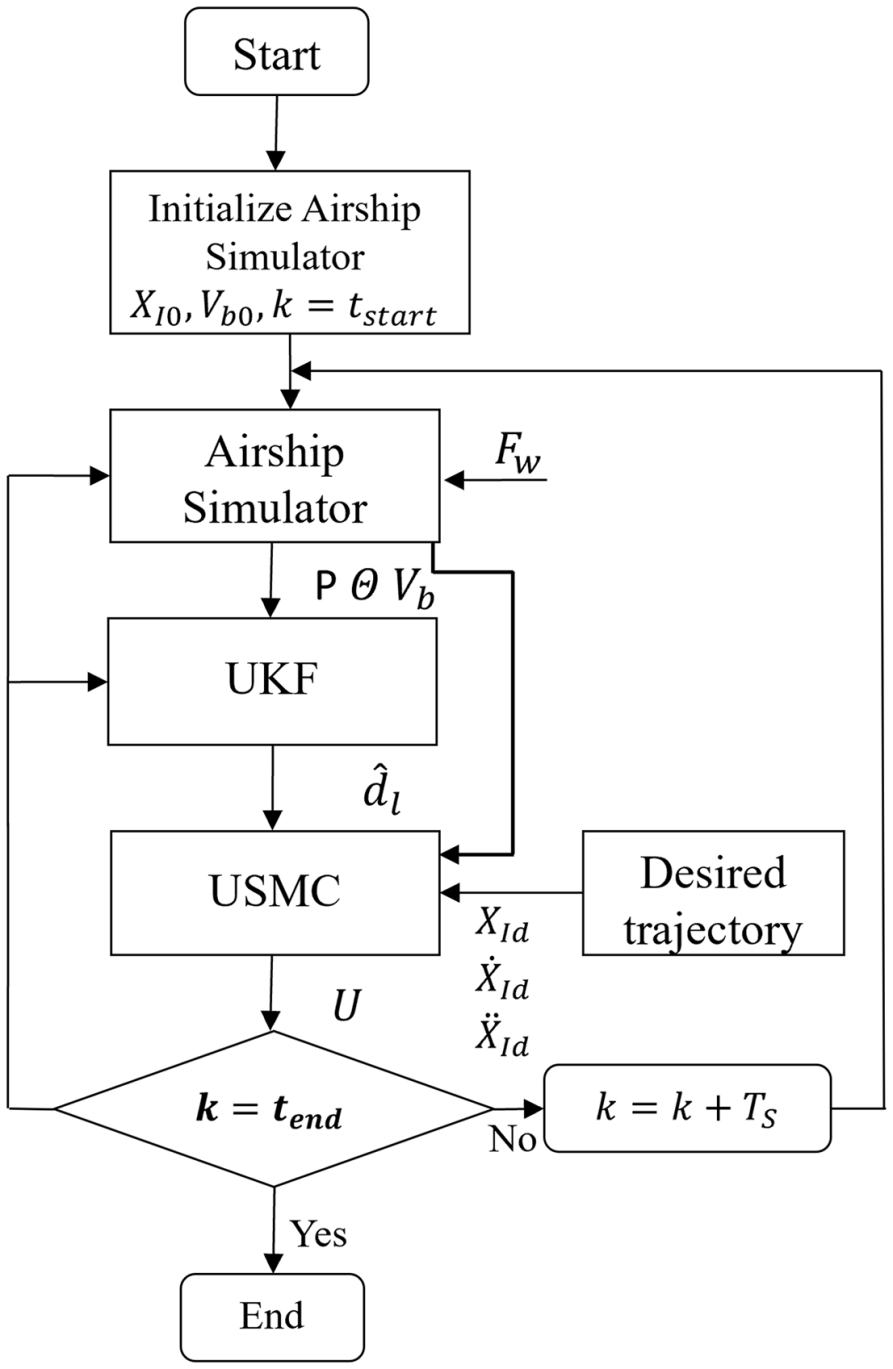
Flow chart for the proposed airship trajectory tracking control algorithm.


Fig 6 shows the flow chart of the proposed algorithm for efficient trajectory tracking of the airship. The airship simulator initializes with the initial values of position, attitude, linear and angular velocities. Table 1 shows the possible initial values for the airship simulator. In the next step, the airship simulator receives control inputs, information on wind disturbance, and airship model parameter variations due to uncertainties. The estimates of position and attitude from the airship simulator are given to the UKF block [31]. UKF block implements an estimation algorithm, based on the airship-modified model given in (22), to estimate the airship state vector and model uncertainty vector. The UKF estimates the airship state and model uncertainty vector. The information regarding states and model uncertainty is given to SMC. The SMC block implements the proposed trajectory tracking control method. It utilizes the estimates of UKF, the desired trajectory, and its derivative to calculate the control action. The stopping criteria of the algorithm depend on the total trajectory time and sampling rate. The relation for $t_{end}$ is:

**Table 1:** Initial conditions for airship simulator.

Symbol	State	Value
${\text{X}}_{\text{0}}$	Position	[−1 m,0 m,67 m]
$\Theta_{\text{0}}$	Attitudes	[0 rad, 0 rad, 0 rad]
$\upsilon_{\text{0}}$	Linear velocities	$\left[\text{0}.\text{5}\text{ m}{\text{s}}^{-\text{1}},\text{0}\text{ m}{\text{s}}^{-\text{1}},\text{0}\text{ m}{\text{s}}^{-\text{1}}\right]$
$\Omega_{\text{0}}$	Angular velocities	$\left[\text{0}\text{ rad}{\text{s}}^{-\text{1}},\text{0}\text{ rad}{\text{s}}^{-\text{1}},\text{0}\text{ rad}{\text{s}}^{-\text{1}}\right]$

$$t_{end}=t_{start}+\frac{desired\;trajectory\;time}{T_{s}}$$ (19)

### 4.2. Unscented Kalman Filter (UKF) design for airship model uncertainties and wind disturbance estimation

Like Extended Kalman Filter (EKF) [31], UKF algorithm is a nonlinear extension of the famous Kalman filter. It is based on the careful selection of sigma points. One can select sigma points using the current mean and state estimation error covariance. These points propagate through the system model to obtain transformed points. This process is known as unscented transform. The method uses the transformed points to obtain the true mean and covariance of the system. In this way, the unscented Kalman filter can approximate any nonlinear system up to the second order of the Taylor series. For the application of the UKF algorithm, it is necessary to represent the nonlinear system in the state-space form. Moreover, it is required to introduce additional state variables for the estimation of unknown parameters or disturbances.

The proposed work introduces six augmented state variables for the estimation of the airship model uncertainty. Vector $d_{l}$, defined in a lumped approach, represents augmented state variables. A constant disturbance model for representing model uncertainty vector $d_{l}$ is used. The constant disturbance model is a simple way of representing a disturbance in a control system as unknown but constant offset added to the system’s dynamics. The airship has a slow dynamic as compared to other arial vehicles; therefore, this assumption is suitable for the airship system. A system model given in equation (2) is modified to apply the UKF algorithm. The augmented vector is:


$$d_{l}=[\begin{array}{cccccc} \Delta F_{u} & {\Delta F}_{v} & \Delta F_{w} & \Delta F_{p} & \Delta F_{q} & {\Delta F}_{r} \end{array}{]}^{\text{T}}$$ (20)

A compact representation for dynamic, aerodynamic, aerostatic, and control input vectors is:


$$F=F_{D}+F_{AS}+F_{AD}+U$$ (21)

Using equations (20), (21), and (2), the airship-modified nonlinear model in state-space form is represented as:


$$\left[\begin{array}{c} {\dot{X}}_{I}\\ \begin{array}{c} {\dot{V}}_{b}\\ {\dot{d}}_{l} \end{array} \end{array}]=[\begin{array}{ccc} R(\Theta ) & O_{\text{6}\times \text{6}} & O_{\text{6}\times \text{6}}\\ O_{\text{6}\times \text{6}} & M^{-\text{1}} & I_{\text{6}\times \text{6}}\\ O_{\text{6}\times \text{6}} & O_{\text{6}\times \text{6}} & O_{\text{6}\times \text{6}} \end{array}][\begin{array}{c} V_{b}\\ \begin{array}{c} F\\ d_{l} \end{array} \end{array}\right]$$ (22)

here, $I_{\text{6}\times \text{6}}$ represents an identity matrix, and $O_{\text{6}\times \text{6}}$ is a matrix with all the elements equal to zero. The modified airship model state vector consists of eighteen state elements as:


$$X=[\begin{array}{ccccc} \text{X} & \Theta & \upsilon & \Omega & d_{l} \end{array}{]}^{T}$$ (23)

The state measurement vector is:


$$Y=CX=[\begin{array}{cc} I_{\text{6}\times \text{6}} & O_{\text{6}\times \text{12}} \end{array}]X$$ (24)

Equation (22) is a continuous-time state-space representation of the airship model. Its compact representation is:


$$\dot{X}=f(X,U)$$ (25)

For the discrete-time UKF algorithm implementation, explicit first-order Euler integration is performed on equation (25). Moreover, it is augmented with process and measurement noise. The discrete-time representation of the model (25) is:


$$X_{k+\text{1}}=IX_{k}+T_{s}f\left(X_{k},U_{k}\right)+W_{p}$$ (26)

$$Y=CX+W_{m}$$ (27)

where $X_{k}$, $T_{s}$, $W_{p}$ and $W_{m}$ denote discrete-time state vector, sampling time, process, and measurement noise vectors, respectively.

UKF algorithm implementation is divided into two steps: prediction and correction [28]

### 4.3. Controller formulation and stability analysis

For USMC, we have the following model available for controller design:


$$\left\{ \begin{array}{c} \begin{array}{c} \begin{array}{c} {\dot{X}}_{I}=R(\Theta )V_{b}\\ {\dot{V}}_{b}=M^{-\text{1}}\left(F_{d}+F_{As}+F_{Ad}+U\right)+{\hat{d}}_{l} \end{array} \end{array} \end{array}\right.\;$$ (28)

**Fig 7:**
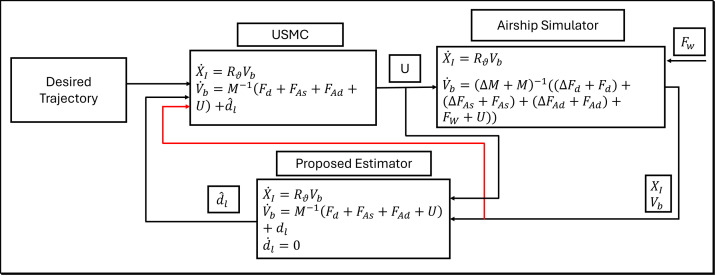
This diagram demonstrates the models used in the airship simulator, USMC, and the estimator.

For clarity, we have incorporated the block diagram shown in Fig 7.

**Assumption 3**. The measurements of airship states are available to the controller. The proposed UKF only estimates the lumped model uncertainty and disturbance vector ${\hat{d}}_{l}$, and acts as a disturbance observer.

**Assumption 4**. Let the estimation error is $\tilde{d_{l}}=d_{l}-{\hat{d}}_{l}$. It is assumed that there exists a positive number $\gamma_{\text{1}}$ such that the estimation error $\left\|{\tilde{d}}_{l}\right\|<\gamma_{\text{1}}$ where $\gamma_{\text{1}}<\gamma$ and further that $\left\|K\right\|>\gamma_{\text{1}}$.

Using the estimate of $d_{l}$ provided by UKF with the extended model given in equation (22), the airship nonlinear model incorporating model uncertainties defined in equations (2), and the sliding surface defined in equation (9). With the given reaching law, the controller equation for USMC results in:


$$U=M{R(\Theta )}^{-\text{1}}\left(-K\text{sign}(S)-\dot{R}(\Theta )V_{b}+{\ddot{X}}_{\text{1}d}-a{\dot{x}}_{e}-R(\theta ){\hat{d}}_{l}\right)-(F_{d}+F_{As}+F_{Ad})$$ (29)

**Theorem 3.** Using the airship model given in equation (2), the sliding surface defined in equation (9), the controller defined in equation (29), and the UKF estimator designed for estimating $d_{l}$ defined in equation (22) will stabilize the closed-loop system. The control law will perform the trajectory tracking task and the tracking error will approach to zero.

**Proof.** Select the Lyapunov function given in the following equation:


$$V=\frac{\text{1}}{\text{2}}S^{T}S>\text{0}$$ (30)

By differentiating equation (30) and expanding the terms, one can obtain:


$$\dot{V}=S^{T}\dot{S}=S^{T}\left[\ddot{x_{e}}+a{\dot{x}}_{e}\right]$$ (31)

Taking the time derivative of equation (8) twice, we get:


$${\ddot{x}}_{e}={\ddot{X}}_{I}-{\ddot{X}}_{Id}$$ (32)

Using equation (2) and simplifying results in:


$${\ddot{x}}_{e}=\dot{R}(\Theta )V_{b}+{R(\Theta )\dot{V}}_{b}-{\ddot{X}}_{Id}=\dot{R}(\Theta )V_{b}+R(\Theta )M^{-\text{1}}\left(F_{d}+F_{As}+F_{Ad}+U\right)+R(\Theta )d_{l}-{\ddot{X}}_{Id}$$ (33)

Substituting equation (33) in equation (31), we obtain:


$$\dot{V}=S^{T}[\dot{R}(\Theta )V_{b}+{R(\Theta )M}^{-\text{1}}\left(F_{d}+F_{As}+F_{Ad}+U\right)+R(\Theta )d_{l}-{\ddot{X}}_{Id}+a{\dot{x}}_{e}]$$ (34)

Using equation (8) and after simplifying, we obtain:


$$\begin{array}{c} \dot{V}=S^{T}[\dot{R}(\Theta )V_{b}+R(\Theta )M^{-\text{1}}(F_{d}+F_{As}+F_{Ad}+M{R(\Theta )}^{-\text{1}}(-Ksign(S)-\dot{R}(\Theta )V_{b}+{\ddot{X}}_{\text{1}d}-a{\dot{x}}_{e}-R(\theta ){\hat{d}}_{l})\\ -\;\left(F_{d}+F_{As}+F_{Ad})\right)+R(\Theta )d_{l}-{\ddot{X}}_{Id}+a{\dot{x}}_{e}] \end{array}$$ (35)

Inserting the relation for control law from equation (29) in equation (35), we obtain:


$$\dot{V}=S^{T}[\dot{R}(\Theta )V_{b}+R(\Theta )M^{-\text{1}}\left(M{R(\Theta )}^{-\text{1}}\left(-Ksign(S)-\dot{R}(\Theta )V_{b}+{\ddot{X}}_{\text{1}d}-a{\dot{x}}_{e}-R(\theta ){\hat{d}}_{l}\right)\right)$$

$$+R(\Theta )d_{l}-{\ddot{X}}_{Id}+a{\dot{x}}_{e}]$$ (36)

$$\dot{V}=S^{T}\left[-K\text{sign}(S)+R(\Theta ){(d}_{l}-{\hat{d}}_{l})\right]<\text{0}$$ (37)

From assumption 3, if the estimation error is bounded by the upper bound $\gamma_{\text{1}}$ and the inequality $\left\|K\right\|>\gamma_{\text{1}}$ exists, we can write:


$$\dot{V}=S^{T}\left[-K\text{sign}(S)+R(\Theta )(\gamma_{\text{1}})\right]<\text{0}$$ (38)

**Remark 4.** The USMC controller designed in equation (28) ensures the Lyapunov stability of an airship system defined by equation (2). The controller ensures that the trajectory tracking error will asymptotically converge to zero within a finite time without satisfying assumption 3.

In the proposed method, UKF estimates $d_{l}$ and provides that information to the SMC. The method ensures the closed-loop stability of the system without the constraints of assumption 3. The proposed method also minimizes the chattering issues that usually come due to the selection of large controller gains for robustness in the conventional SMC. The performance of the proposed trajectory tracking control scheme is evaluated using a nonlinear simulator designed for UETT airship.

## 5. Results and discussion

For the verification of controller performance, we performed numerical simulations using a variable step RK (Runga-Kutta) method in MATLAB/Simulink R2019b environment. The software operates on a computer with a CPU frequency of 2.5 GHz. The sampling time used for the simulation is 0.002 s. An experimental UETT airship 6-DOF nonlinear model is developed. The UETT airship mass matrix, aerodynamic model, aerostatic model, and control input matrix parameters are given in [32,33].

The initial conditions used for the airship simulator are given in Table 1. The initial conditions used for UKF are obtained while doing the engineering analysis of the problem at hand. The filter tuning is discussed in subsection 5.1.

### 5.1. Selection of filter parameters

The selected filter parameters are given below:

#### 5.1.1. Initial state error covariance matrix.

$P_{\text{0}}$corresponds to the initial value of state error covariance. Let it is:


$$\sqrt{P_{\text{0}}}=\text{diag}\left(\sqrt{P_{\text{01}}}\;,\ldots ,\sqrt{P_{\text{18}}}\right)$$ (39)

The first twelve state elements are the airship position [x, y, z], attitude [φ , θ, ψ], linear [u, v, w], and angular velocities [p, q, r]. It is assumed that the estimates of airship position, attitude, and linear and angular velocities are available. Such an assumption can be made practically as off-the-shelf solutions involving sensor fusion techniques are available for their estimation [34].

Current research assumes the estimates available using the solution given by [34]. Therefore, the first twelve elements of *P*_0_ are selected based on the possible state error in the estimates of sensor fusion based on EKF [34].

**Table:** 

$\begin{array}{c} \sqrt{P_{01}} \end{array}$	2.8 m	$\begin{array}{c} \sqrt{P_{07}} \end{array}$	1.1 ms^-1^
$\sqrt{P_{\text{0}\text{2}}}$	0.9 m	$\sqrt{P_{\text{0}\text{8}}}$	1.9 ms^-1^
$\sqrt{P_{\text{0}\text{3}}}$	0.01 m	$\sqrt{P_{\text{0}\text{9}}}$	1.1 ms^-1^
$\sqrt{P_{\text{0}\text{4}}}$	0.01 rad	$\sqrt{P_{\text{1}\text{0}}}$	0.01 rads^-1^
$\sqrt{P_{\text{0}\text{5}}}$	0.01 rad	$\sqrt{P_{\text{1}\text{1}}}$	0.01 rads^-1^
$\sqrt{P_{\text{0}\text{6}}}$	1.7 rad	$\sqrt{P_{\text{1}\text{2}}}$	0.09 rads^-1^

The last six elements of the state error covariance matrix ($\sqrt{P_{\text{1}\text{3}}}$ to $\sqrt{P_{\text{1}\text{8}}}$) represent the initial state error covariance of the augmented states. The augmented states represent the rate of change of lumped uncertainty terms. The lumped uncertainty terms capture the cumulative effects on the airship dynamic accelerations due to model uncertainties and wind disturbance. So, the variations in dynamic accelerations due to the following three reasons will be checked:

- Mass matrix parameter uncertainty
- Aerodynamic model uncertainty
- Wind disturbance

**Fig 8:**
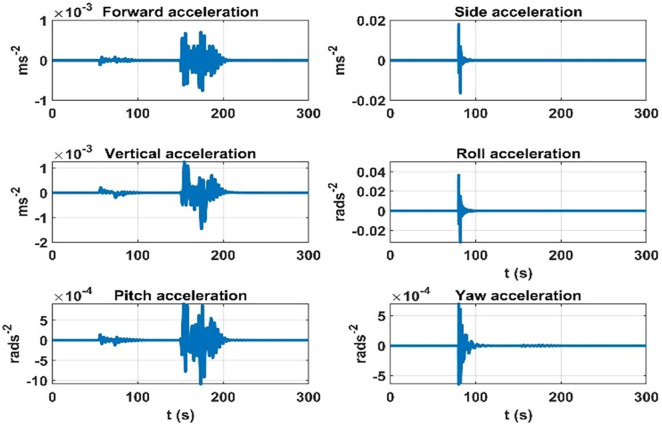
Rate of change of the effect on dynamic accelerations due to mass matrix variation parameters.

First, an experiment is devised where during the airship flight, the change in the air-ship CG is introduced, and the rate of change in dynamic accelerations due to the change in CG is plotted. This experiment considers the 2.3 ms^-1^ speed of the airship. The results are shown in Fig 8.

**Fig 9:**
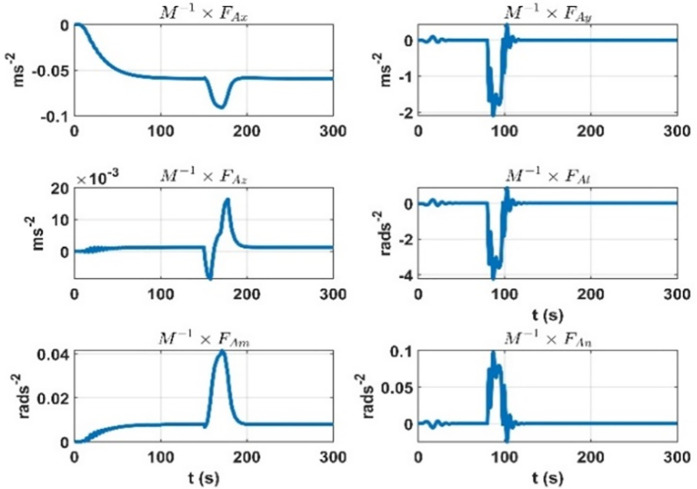
Rate of change of the effect on dynamic accelerations due to aerodynamic forces.

For quantifying the effect due to the aerodynamic model on dynamic accelerations, the same simulation case is considered. Thruster, rudder, and elevator control inputs at five, eighty, and one-fifty seconds of the simulation run are applied, respectively. The purpose of this investigation is to quantify the effects of dynamic accelerations due to aerodynamic forces and torques. The rate of change of accelerations due to the aerodynamic model is summarized in Fig 9.

**Fig 10:**
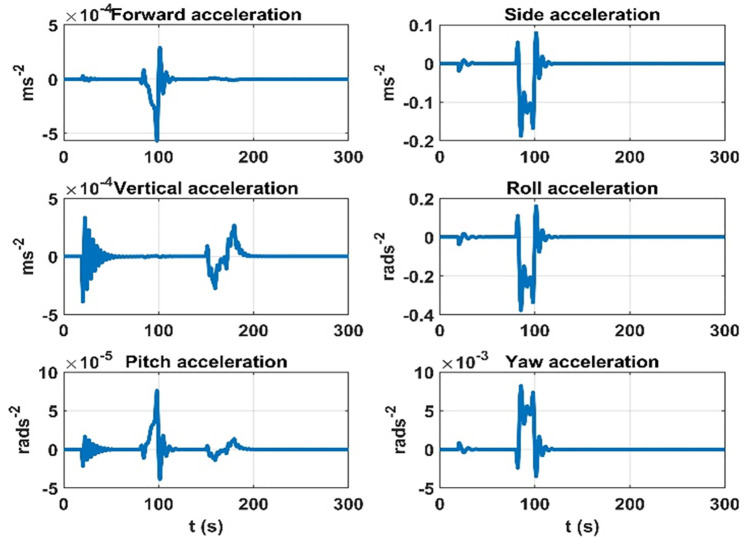
Rate of change of the effect on dynamic accelerations due to wind forces.

The wind is added to the simulation after 20 seconds of the aerodynamic flight of the airship. Wind forces are not used in calculating velocities at the body axes of the airship. However, its possible effect is quantified based on the angle of attack and sideslip angle of the airship. The effect of wind forces is calculated in terms of possible acceleration changes and their rates. This is done while the airship executes straight-level flight, maneuvering at some bank angle and climbing at some pitch angle. The results are given in Fig 10. Therefore, the elements ($\sqrt{P_{\text{1}\text{3}}}$ to $\sqrt{P_{\text{1}\text{8}}}$) of the initial variance matrix are given below. They represent the maximum rate of change in dynamic accelerations that occur due to lumped model uncertainties and wind disturbance.

**Table:** 

$\begin{array}{c} \sqrt{P_{13}} \end{array}$	2.8 ms^-2^	$\begin{array}{c} \sqrt{P_{16}} \end{array}$	0.01 rad s^-2^
$\sqrt{P_{\text{1}\text{4}}}$	0.9 ms^-2^	$\sqrt{P_{\text{1}\text{7}}}$	0.01 rad s^-2^
$\sqrt{P_{\text{1}\text{5}}}$	0.01 ms^-2^	$\sqrt{P_{\text{1}\text{7}}}$	1.7 rad s^-2^

#### 5.1.2 Process covariance matri𝐱 Q.

Let the Q matrix is given as:


$$\sqrt{Q}=\text{diag}\left(\sqrt{q_{\text{01}}}\;,\ldots ,\sqrt{q_{\text{18}}}\right)$$ (40)

The first six terms ($\sqrt{q_{\text{0}\text{1}}}$ to $\sqrt{q_{\text{0}\text{6}}}$) of the process covariance matrix are associated with the kinematic modelling equations of the model. These modelling equations have no error. So, we select the small values for these elements of the process noise covariance matrix as it shows our trust in this part of the model. So, the first six diagonal terms of the matrix are:

**Table:** 

$\begin{array}{c} \sqrt{q_{01}} \end{array}$	0.1 m/sample	$\begin{array}{c} \sqrt{q_{04}} \end{array}$	0.1 rad/sample
$\sqrt{q_{\text{0}\text{2}}}$	0.1 m/sample	$\sqrt{q_{\text{0}\text{5}}}$	0.1 rad/sample
$\sqrt{q_{\text{0}\text{3}}}$	0.1 m/sample	$\sqrt{q_{\text{0}\text{6}}}$	0.1 rad/sample

**Fig 11:**
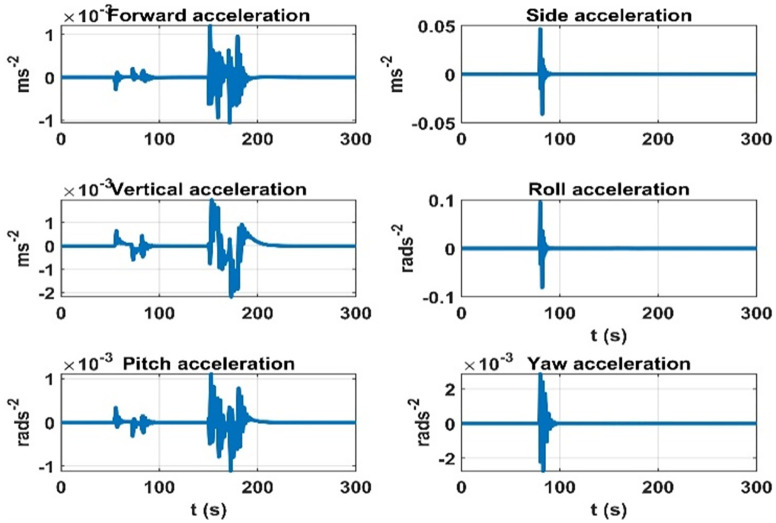
Accelerations due to the mass matrix parameters variation at 5.5 ms^−^^1^ of the airship speed.

**Fig 12:**
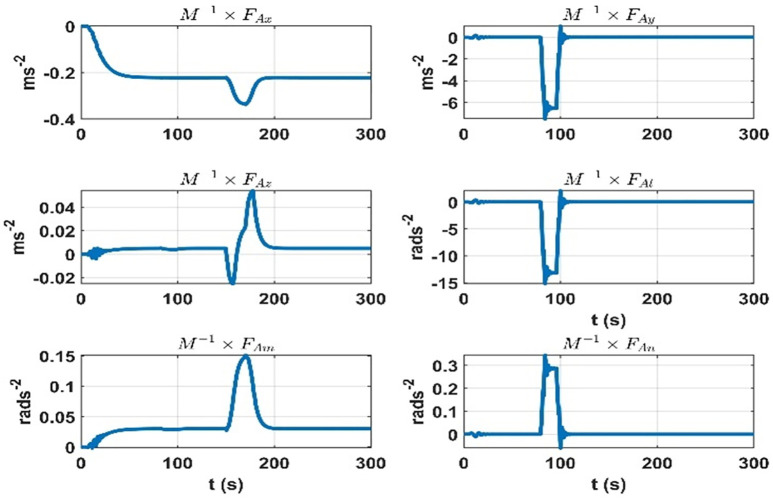
Accelerations are due to aerodynamic forces over time when the rudder and elevator deflections are applied at the 5.5 ms^-1^ speed of the airship.

**Fig 13:**
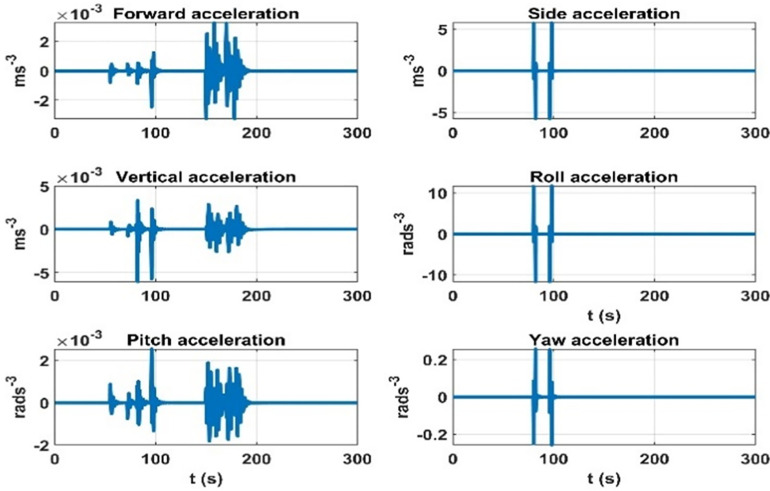
Rate of change of accelerations due to mass matrix parameters variation at 5.5 ms^-1^ speed of the airship.

The terms seven to twelve ($\sqrt{q_{\text{0}\text{7}}}$ to $\sqrt{q_{\text{1}\text{2}}}$) depend on the effect on dynamic accelerations due to the lumped uncertainty and disturbance. As discussed, the initial state error covariance matrix depends on the mass matrix parameters variations, aerodynamic model, and disturbances. To sum up the mass matrix parameter variation effects, the same experiment is performed as discussed in the initial state error covariance matrix section at the airship speed of 5.5 ms^*-*1^. The results are given in Fig 11. Next, for the accelerations due to aerodynamic forces and torques, the airship is subjected to rudder, elevator, and thruster inputs. The speed of airship during the experiment was 5*.*5 ms^*-*1^. The results are given in Fig 12. The changes in dynamic accelerations due to wind forces are given in Fig 13.

Considering these results, the terms seven to twelve ($\sqrt{q_{\text{0}\text{7}}}$ to $\sqrt{q_{\text{1}\text{2}}}$) of the Q matrix are given below. They represent the maximum rate of change in dynamic accelerations which occur due to lumped model uncertainties and wind disturbance.

**Table:** 

$\begin{array}{c} \sqrt{q_{07}} \end{array}$	0.6 m/sample^2^	$\begin{array}{c} \sqrt{q_{10}} \end{array}$	30 rad/sample^2^
$\sqrt{q_{\text{0}\text{8}}}$	15 m/sample^2^	$\sqrt{q_{\text{1}\text{1}}}$	0.2 rad/sample^2^
$\sqrt{q_{\text{0}\text{9}}}$	0.05 m/sample^2^	$\sqrt{q_{\text{1}\text{2}}}$	0.6 rad/sample^2^

**Fig 14:**
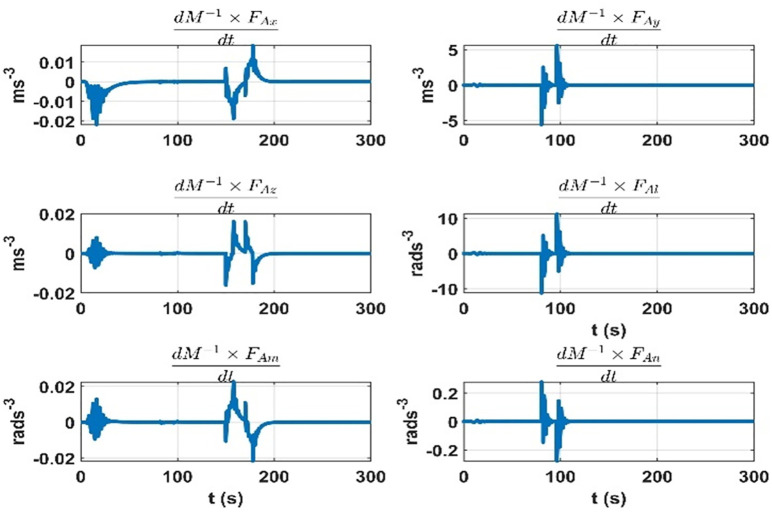
Rate of change of accelerations due to the aerodynamic forces at 5.5 ms^−^^1^ speed of the airship.

**Fig 15:**
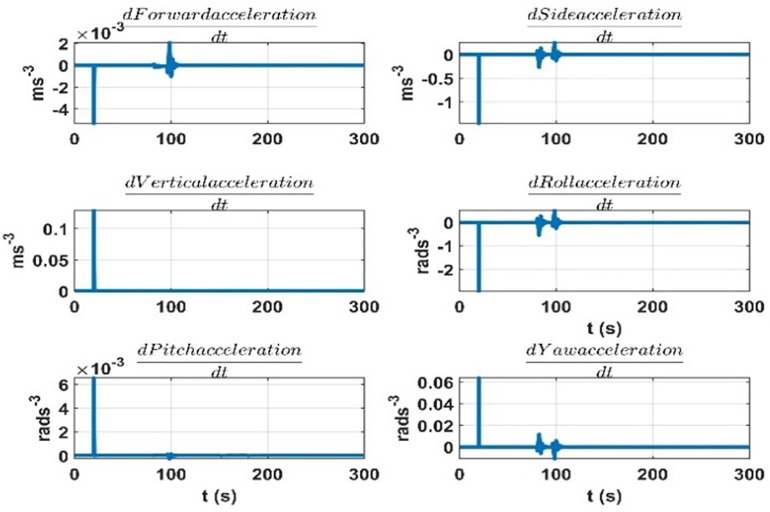
Rate of change of accelerations due to the wind forces at 5.5 ms^-1^ speed of the airship.

**Fig 16:**
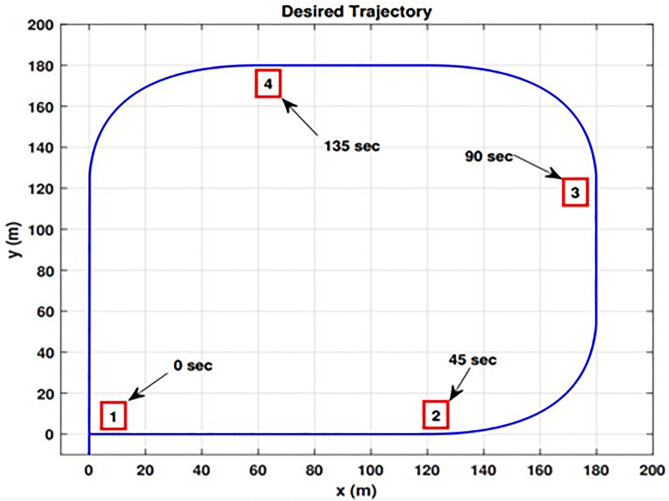
Desired trajectory.

The terms thirteen to eighteen ($\sqrt{q_{\text{1}\text{3}}}$ to $\sqrt{q_{\text{1}\text{8}}}$) depend on the error in the modelling of augmented states. Since the augmented states represent the rate of change of the lumped uncertainty vector, we consider the possible variations of this vector as we did for the initial state error covariance matrix. We repeat the experiments for 5*.*5 ms^*-*1^ speed of the airship. The rate of change of lumped uncertainty vector due to mass matrix parameters variations, aerodynamic forces, and wind disturbance is shown in Figs 14–16. Therefore, the terms thirteen to eighteen ($\sqrt{q_{\text{1}\text{3}}}$ to $\sqrt{q_{\text{1}\text{8}}}$) of the *Q* matrix are given below. They represent the maximum rate of change in dynamic accelerations that occur in Figs 14–15.

**Table:** 

$\begin{array}{c} \sqrt{q_{13}} \end{array}$	0.05 m/sample^3^	$\begin{array}{c} \sqrt{q_{16}} \end{array}$	10 rad/sample^3^
$\sqrt{q_{\text{1}\text{4}}}$	5 m/sample^3^	$\sqrt{q_{\text{1}\text{7}}}$	0.03 rad/sample^3^
$\sqrt{q_{\text{1}\text{5}}}$	0.03 m/sample^3^	$\sqrt{q_{\text{1}\text{8}}}$	0.2 rad/sample^3^

#### 5.1.3 Measurement noise covariance matrixR.

Let the R matrix is:


$$\sqrt{R}=\text{diag}\left(\sqrt{r_{\text{01}}}\;,\ldots ,\sqrt{r_{\text{18}}}\right)$$ (41)

The measurement noise covariance matrix depends on the possible error in the sensor readings. In our case, we are not directly taking measurements from the sensors, rather we are relying on the EKF-based sensor fusion algorithm. Therefore, in this case, the measurement noise is the error in the estimate of the sensor fusion algorithm [32].

**Table:** 

$\begin{array}{c} \sqrt{r_{01}} \end{array}$	2.8 m	$\begin{array}{c} \sqrt{r_{07}} \end{array}$	1.1 ms^-1^
$\sqrt{r_{\text{0}\text{2}}}$	0.9 m	$\sqrt{r_{\text{0}\text{8}}}$	1.9 ms^-1^
$\sqrt{r_{\text{0}\text{3}}}$	0.01 m	$\sqrt{r_{\text{0}\text{9}}}$	1.1 ms^-1^
$\sqrt{r_{\text{0}\text{4}}}$	0.01 rad	$\sqrt{r_{\text{1}\text{0}}}$	0.01 rads^-1^
$\sqrt{r_{\text{0}\text{5}}}$	0.01 rad	$\sqrt{r_{\text{1}\text{1}}}$	0.01 rads^-1^
$\sqrt{r_{\text{0}\text{6}}}$	1.7 rad	$\sqrt{r_{\text{1}\text{2}}}$	0.09 rads^-1^

### 5.2 Controller performance

This section covers the simulation results for the performance evaluation of the proposed method using the simulation environment developed for the experimental UETT airship. We evaluate the performance of the proposed trajectory tracking controller by tracking the square trajectory under model uncertainties and disturbances. The outdoor experimental area for the UETT airship consists of cricket and football ground fields. In this simulation experiment, we have considered 180 m x 180 m of the field area. The edges of the desired trajectory are not considered sharp because of the dynamic restrictions of the UETT airship and to keep the desired trajectory and its consecutive derivatives smooth. Four observation points are considered on the desired trajectory, as highlighted in Fig 16. The first point indicates the start of the desired trajectory. The second indicates the first maneuver, where the airship takes ninety degrees of turn. On the third and fourth observation points, it completes the 270 degrees of turning motion, as shown in Fig 16. The initial conditions used for the airship model are given in Table 1. The initial conditions for the estimator are given in Table 2. The state error covariance matrix, process noise covariance matrix, and measurement noise covariance matrices are given in Table 2.

**Table 2:** Initial conditions for the estimator.

Symbol	State	Value
${\text{X}}_{\text{0}}$	Position	$[-\text{2}\text{ m},\text{2}\text{ m},\text{7}\text{0}\text{ m}{]}^{T}$
$\Theta_{\text{0}}$	Attitudes	$[\text{0}\text{ rad},\;\text{0}\text{ rad},\;\text{0}\text{ rad}{]}^{T}$
$\upsilon_{\text{0}}$	Linear velocities	${\left[\text{2}\text{ m}{\text{s}}^{-\text{1}},\text{0}\text{ m}{\text{s}}^{-\text{1}},\text{0}\text{ m}{\text{s}}^{-\text{1}}\right]}^{\text{T}}$
$\Omega_{\text{0}}$	Angular velocities	${\left[\text{0}.\text{2}\text{ rad }{\text{s}}^{-\text{1}},\text{0}.\text{3}\text{ rad}{\text{ s}}^{-\text{1}},\text{0}.\text{1}\text{ rad }{\text{s}}^{-\text{1}}\right]}^{T}$
$d_{l}$	uncertainty vector	$[\begin{array}{cccccc} \text{0} & \text{0} & \text{0} & \text{0} & \text{0} & \text{0} \end{array}{]}^{T}$
Q	Process noise covariance	diag(0.1, 0.1, 0.1, 0.1, 0.1,0.1, 0.6, 15, 0.05, 30, 0.2, 0.6, 0.05, 5, 0.03, 10, 0.03,0.2)
R	Measurement noise covariance	diag (2.8, 0.9, 0.01, 0.01, 0.01, 1.7, 1.1, 1.9, 1.1, 0.01,0.01, 0.09)
$P_{\text{0}}$	State error covariance	diag(2.8, 0.9, 0.1, 0.1, 0.1,1.7, 1.1, 1.1, 1.7, 0.01, 0.01, 0.09, 2.8, 0.9, 0.1, 0.1, 1.7)

The same gains for both controllers are used to demonstrate the benefits of available estimates for uncertainty and disturbance. The gains are given in Table 3. The gain matrix α shows the convergence properties of error when the controller is in the sliding phase. The longitudinal dynamics of airships are slow compared to the lateral dynamics. So, accordingly, fast convergences can be expected for lateral motion variables. Therefore, the gains for lateral variables are high compared to the longitudinal ones. The gain matrix K is related to the robust characteristics of the controller. The values in the matrix depend on the model uncertainty and available actuation. Higher values may achieve a robust response at the cost of high control effort which is undesirable in the case of the airship. High gain values may lead to actuator saturation that may cost system stability. The small controller gains also reduce the chattering phenomena.

**Table 3:** Sliding mode controller gains.

Symbol	State
α	diag(2, 50, 2, 50, 2, 50)
K	diag(1, 1, 1, 1, 1, 1)

**Fig 17:**
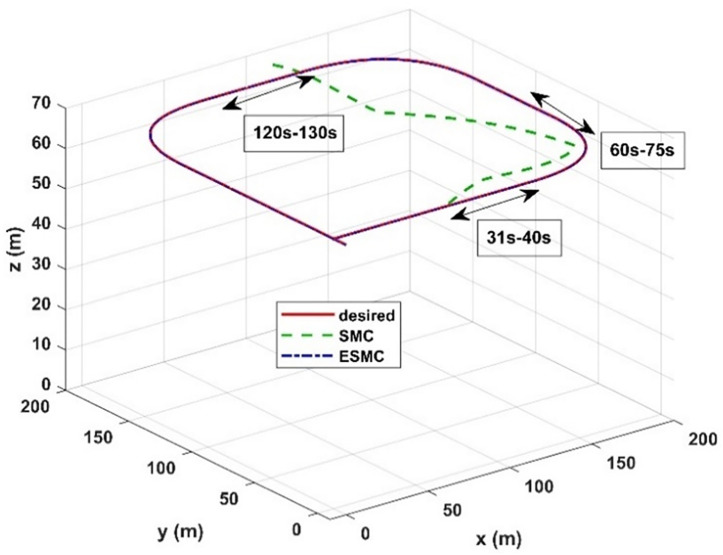
Trajectory tracking performance of SMC and USMC methods.

The trajectory tracking of the airship under both control methods (SMC and USMC) is evaluated, and the results are given in Fig 17. During the simulation at different time intervals, model parameters variation, and wind disturbance with wind gusts are applied. During an interval of 31 seconds to 40 seconds, changes in the airship aerodynamic model are introduced. Between 60 seconds to 75 seconds, the center of gravity of the airship is varied and wind disturbances are applied between 120 seconds to 130 seconds. It can be seen in Fig 20 that the proposed control method successfully tracks the desired trajectory while the SMC method loses closed-loop stability when wind disturbance is applied. The tracking performance of both controllers is the same till a time interval of 30 seconds of the aerodynamic flight of the airship.

**Fig 18:**
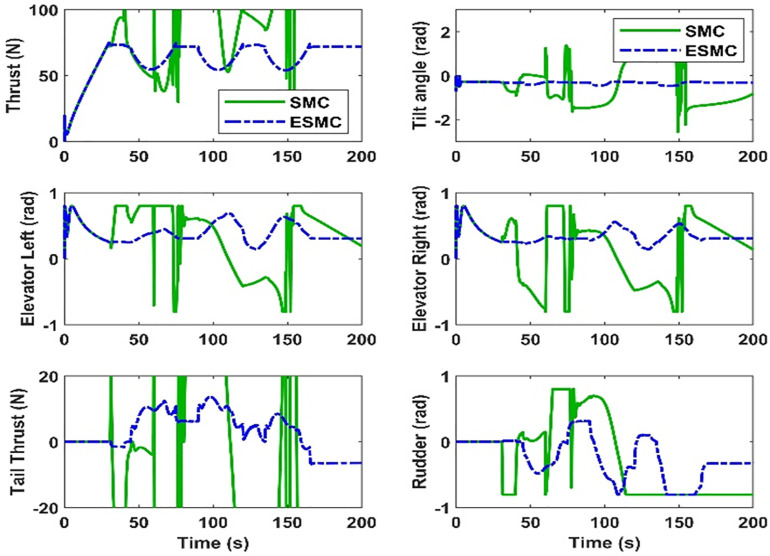
Control efforts (1) The total thrust required to keep the positive value of forwarding velocity (ii) The tilt angle of the thrusters that are required for vector thrusting (iii) Left elevator deflection (iv) Right elevator deflection (v) Tail thrust value (vi) Rudder deflection. The tail thruster and rudders control the airship’s side velocity and yaw rate. The airship roll is controlled by left and right elevators. The main vector thrusting is required to control the airship’s forward velocity, pitch rate, and vertical velocity of the airship.

**Fig 19:**
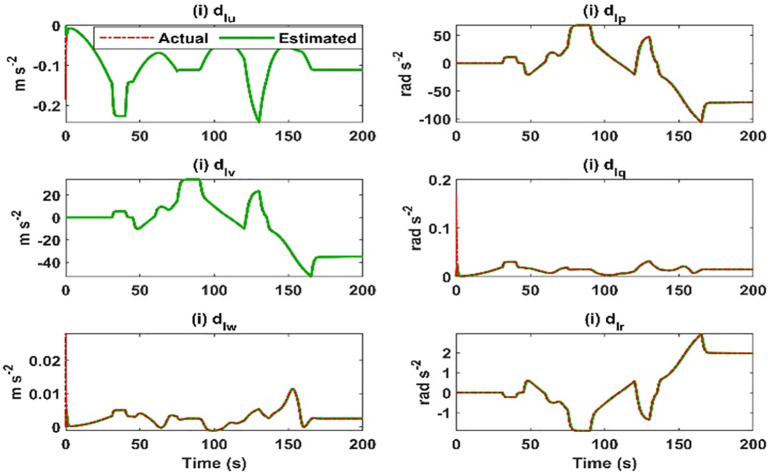
Lumped model uncertainty vector estimation results.

**Fig 20:**
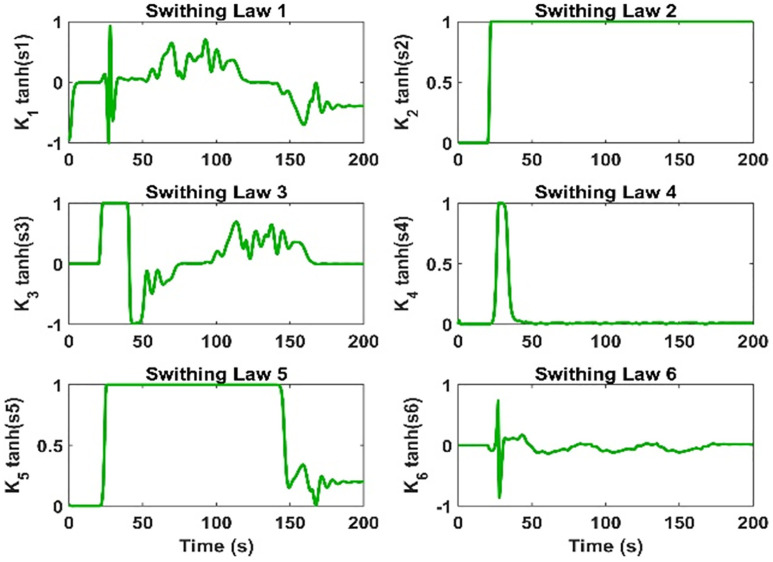
The switching of the proposed controller.

The SMC method performs well when the dynamic model information is accurately known. However, after 30 seconds, the SMC method tracks trajectory with a large estimation error. This shows how the uncertain dynamics of the airship affect the performance of the SMC. The tracking error for the proposed control method is small and the airship remains on the desired path. After 60 seconds, when variation in the airship aerodynamic model is introduced, the SMC method loses track and closed-loop stability is lost after 125 seconds of the simulation run.

The control efforts calculated by both controllers are given in Fig 18. In the case of the USMC method, the control efforts are smooth and fulfil the requirements of trajectory tracking. However, for the SMC method, the control efforts suffer from the saturation problem, and eventually, the controller loses stability. The estimation of lumped model uncertainty and wind disturbances are given in Fig 19. The switching properties of the proposed control method are given in Fig 20.

## 6. Conclusion

An unscented Kalman filter is used in this work to provide accurate estimates for unknown model parameters. A hybrid control method for known and unknown model parameters coupled with a sliding mode controller enables the airship to track the desired trajectory accurately.

UKF-based SMC improves robustness by designing a nonlinear estimation procedure for wind disturbances and airship model uncertainties. The convergence and stability analysis of the proposed method proves that the hybrid USMC technique is convergent and closed-loop stable. Extensive simulations are performed for the performance evaluation of the controller under the mass matrix parameter variations, aerodynamic forces and torque variations, and wind disturbances. The results show that the USMC method is efficient and effective for steering the airship along the reference trajectory for an infinite time. Further, USMC improves the robust characteristics of the conventional SMC method and minimizes the chattering.

From the information of the hybrid sensor system, position, altitude, and known dynamic model parameters of the airship are estimated. These estimates allow investigation of the effects induced by wind instead of measuring wind velocities by costly instrumentation.

The proposed method is realizable, cost-effective, and understandable. It allows variation in model parameters without compromising the performance of trajectory tracking of the airship. Compared to intelligent estimation solutions such as Neural Networks, the proposed method is a generic solution to the airship trajectory tracking control problem. It can be used for trajectory tracking control problems for all types of UAVs.
